# BMAL1 plays a critical role in the protection against cardiac hypertrophy through autophagy in vitro

**DOI:** 10.1186/s12872-022-02822-3

**Published:** 2022-08-22

**Authors:** Lei Yu, Lei Ren, Linchang Dong

**Affiliations:** Institute of Physical Education, Jiangsu Second Normal University, 6 Xinhe West Rd, Nanjing, 211200 Jiangsu China

**Keywords:** BMAL1, Cardiac hypertrophy, Oxidative stress, Autophagy

## Abstract

**Background:**

Heart disease could result from a malfunction in the core clock gene BMAL1, according to studies conducted on animals and humans in vitro and in vivo. However, in pathological conditions, the role of BMAL1 was not clear. In the present study, we identified a potential link between BMAL1 and cardiac hypertrophy.

**Methods:**

Primary cultured neonatal rat cardiomyocytes were stimulated by Ang II. Cardiomyocytes immunofluorescence analysis was performed to observe the cell size. RT-PCR and Western blot were used to find out the gene and protein expression. Cell apoptosis was measured by TUNEL staining. The Elisa assay was performed which determine the release of cytokines led to the activation of cardiac fibro-blasts in cell-free supernatants. Furthermore, gain- and loss-of-function studies revealed that BMAL1 has an effect on Ang II-induced cardiac hypertrophy.

**Results:**

We found that Ang II-induced cardiac hypertrophy as a result BMAL1 expression was reduced. However, overexpression of BMAL1 could prevent Ang II-induced hypertrophy. Additionally, although BMAL1 overexpression in hypertrophic cardiomyocytes could not prevent hypertrophy, it did reduce the apoptosis of hypertrophic cardiomyocytes after Ang II had induced it. In addition, BMAL1 knockdown did not aggravate Ang II-induced hypertrophy but accelerated its development. Finally, BMAL1 overexpression significantly resisted the effects of Ang II on oxidative stress, autophagy and, cardiac fibrosis in cardiomyocytes.

**Conclusions:**

Our results showed that overexpression of BMAL1 effectively resisted cardiac hypertrophy induced by Ang II. Our findings provided a novel potential target for the treatment of cardiac hypertrophy.

**Supplementary Information:**

The online version contains supplementary material available at 10.1186/s12872-022-02822-3.

## Introduction

Cardiac hypertrophy (CH) was a compensatory response of the heart to overstimulation. However, prolonged compensation might lead to heart failure, which was the leading cause of death from cardiovascular disease [1]. The mechanism of CH had not been fully elucidated, but more and more studies showed that it was closely related to gene transcription, calcium regulation, inflammation, metabolism, protein synthesis, oxidative stress and autophagy [2–9]. In particular, the molecular and pathway mechanisms associated with these processes were considered to be targets for the treatment of CH.

Recent studies revealed that disruption of circadian rhythms in humans and animals was a risk factor for many diseases such as obesity, type 2 diabetes, and cardiovascular diseases [10–12]. The circadian clock is adapted to the day-night cycle of the earth and is a fundamental mechanism of evolutionary conservation in living organisms [13]. At the cellular level, circadian rhythm was composed of transcriptional-translational feedback loops (TTFLs) which were based on delayed negative feedback. TTL core protein components include transcription factors BMAL1 (Arntl), CLOCK, and NPAS2 [14]. These transcription factors regulated many physiological functions directly or indirectly by driving the rhythmic expression of lots of clock-controlled genes.

Especially for BMAL1, the BMAL1 deficient mice showed symptoms of dilated cardiomyopathy, a disorder of left ventricular dilation and contraction [15]. Also, a BMAL1 knockout human embryonic stem cell (hESC) model showed that BMAL1 deficient hESC-derived cardiomyocytes exhibited typical phenotypes of dilated cardiomyopathy including attenuated contractility, calcium dysregulation, and disorganized myofilaments [16]. These results suggested that dysregulation of BMAL1 might contribute directly to abnormality cardiomyopathy. However, the role of BMAL1 in the development and prevention of cardiomyopathy was not well understood.

In this study, primary cultured neonatal rat cardiomyocytes (NRCMs) were stimulated by Ang II, and we found that after Ang II treatment, the expression of BMAL1 significantly reduced in NRCMs. Furthermore, gain-and loss-of-function studies revealed that BMAL1 could resist the effect of Ang II on NRCMs. Finally, we further discovered that BMAL1-prevented CH was partially dependent on myocardial autophagy.

## Materials and methods

### Cell cultures and treatments

Five neonatal rats were obtained from Nanjing University's Model Animal Research Center; the animals were not bred and were sterilized directly with alcohol immersion before being killed by cervical dislocation. The heart was quickly removed, and the ventricles were washed three times with PBS before being incubated for 15 min with 0.125% trypsin–EDTA (2520-072; GIBCO, USA). The heart was then digested four times for 15 min each using PBS containing 0.125% trypsin–EDTA. The digested suspension was transferred to a 50 ml centrifuge tube containing 15 ml of primary culture neutralizing fluid to stop the trypsin activity. The cells were centrifuged with 250 × G for 8 min and then resuscitated with DMEM/F12 (C11330; GIBCO) medium containing 15% fetal bovine serum (10099; Gibco) for 2 h. The cells were then inoculated in 6-well plates with 5 × 10^5^ cell densities and incubated with 5% CO_2_ at 37℃ for 48 h. The culture media was DMEM/F12 containing 15% fetal bovine serum and 1% 5-bromo-2′-deoxyuridine. To simulate in vitro myocardial hypertrophy, the cultured cells were starved for 12 h in serum-free DMEM/F12 medium and then stimulated with Ang II (a9525; Sigma-aldrich, USA, 1 μm) for another 24 h.

### Cardiomyocytes immunofluorescence analysis

Cell surface area was measured by immunofluorescence staining as previously described [17]. Myocardial cells were first immobilized with 3.7% formaldehyde and then sealed with a PBS solution containing 0.1% Triton X-100 at 4 °C overnight. After that, the cells were stained with α-actinin (3134; Cell Signaling Technology; USA) and subsequently incubated with Alexa Fluor 488 (green) secondary antibodies (1:200) for 60 min at 37 °C. The nuclei were re-stained with 4,6-diamidino-2-phenyl (DAPI). The cell surface area was measured with image-pro plus version 6.0. Under 40 × visual field, 6–10 microscopic visual fields were randomly selected and 5 cells were counted in each visual field (30–50 cells in each group). AOI was used to automatically track cell boundaries, and cell area conversion was accomplished using the COUNT/SIZE tool on a scale of equal magnification.

### RT-PCR

In order to detect the mRNA expression of core clock genes, markers related to CH, and genes related to oxidative stress and autophagy, according to the instructions of TRIzol Kit (15596-026, invitrogen, USA), the total mRNA was extracted. Subsequently, the corresponding Oligo (DT) primers were reverse-transcribed into cDNA by cDNA Synthesis Kit (4897030001; Roche; Switzerland) and amplified by real-time quantitative PCR (Light Cycler 480; Roche). The primer of β-Actin was included for normalization. A complete list of PCR primers was shown in Additional file 1: Table S1.

### Western blot

The expression of BMAL1 protein and proteins related to oxidative stress and autophagy was determined by the WB technique as previously described [17]. Total proteins were extracted by using RIPA lysis buffer supplemented by a protease inhibitor cocktail (Complets, Roche). The equivalent (50 μg) protein was transferred onto the PVDF membrane (IPFL00010; Millipor; USA) after 10% SDS-PAGE electrophoresis. Then, the membranes were blocked with 5% skimmed milk at room temperature and incubated overnight at 4 °C with primary antibodies. The antibody reagents were BMAL1 (ab3350; Abcam; USA,), β-Actin (AP0060; Abcam), Gp91phox (ab129068, Abcam) p67phox (3923, Cell Signaling technology), SOD2 (ab38155, Abcam), HO-1 (ab13243, Abcam), LC3 (12741, CST, USA), P62 (23214, CST), ATG 5,12 (AAM79-1, AbD Serotec, UK), ATG 7(2631, Cell Signaling technology) and Beclin1 (3738, Cell Signaling technology). HRP-conjugated secondary antibodies were then applied to bind and visualize the primary antibodies. Finally, images were obtained by Odyssey Infrared Imaging System (LI-COR Biosciences, USA) to quantify protein expression.

### TUNEL staining

A fluorometric TUNEL detection kit (Genecopoeia, USA) was used to detect apoptotic DNA strand breaks as previously described [18]. The NRCMs were fixed with 4% neutral buffered formaldehyde in PBS (pH 7.4) at 25 °C for 30 min, permeated with 50 μg/ml proteinase K at 25 °C for 15 min, and incubated with the labeling reaction mixture in a humidified chamber at 37 °C for 1 h. The cells were then processed with a standard immunocytochemical staining procedure to incubate with antibody against DAPI (Invitrogen, CA). Finally, an Olympus fluorescence microscope (BX51, Olympus, Japan) was used to capture the images, and the ratio of TUNEL positive nuclei in total (DAPI positive nuclei) was computed to express the cells apoptosis.

### Enzyme linked immunosorbent assay (Elisa)

The concentration of cytokine in cell-free supernatants were measured by the Elisa assay and magnetic rat high sensitivity cytokine detection kits (Yancheng Jiumu Bioengineering Institute, China), which included TGF-β1 (MM-0033M1), TNF-α (MM-0132M1), IL-6 (MM-0163M1), IL-18 (MM-0139H1) and IL-1β (MM-0181H1) and the process was determined strictly following the instructions of the manufactures.

### Recombinant viral vectors and infection

To knock down BMAL1 expression, NRCMs were cultured with DMEM/F12 containing 15% fetal bovine serum and 1% 5-bromo-2′-deoxyuridineand transfected with either Bmal1 shRNA or scramble shRNA for 24 h followed by 24-h AngII treatment. We performed transfection by using Lipofectamine 3000 reagent (Invitrogen, USA) according to the manufacturer’s instructions.

To induce BMAL1 overexpression, we transduced adenoviruses encoding either the BMAL1 CDS domain or its negative controls at a dose of 1 × 10^10^ plaque-forming units (PFU). Detailed sequences were listed in Additional file 1: Table S1. NCRMs were infected with the virus at a multiplicity of infection of 100 for 8 h before or after 24-h AngII treatment.

### Statistical analysis

All data were analyzed with Origin 8 (MicroCal Software, USA) and statistical analysis results were expressed as means ± standard errors of the means (SEM). After having checked the normality of data (normality test, with a 5% confidence interval), a one-way analysis of variance (ANOVA) was used to evaluate differences between multiple groups, and student’s t-test was employed for statistical comparison between two groups, *P* values of < 0.05 were considered to be significant.

## Results

### CH led to the decrease of BMAL1 expression

**Fig. 1:**
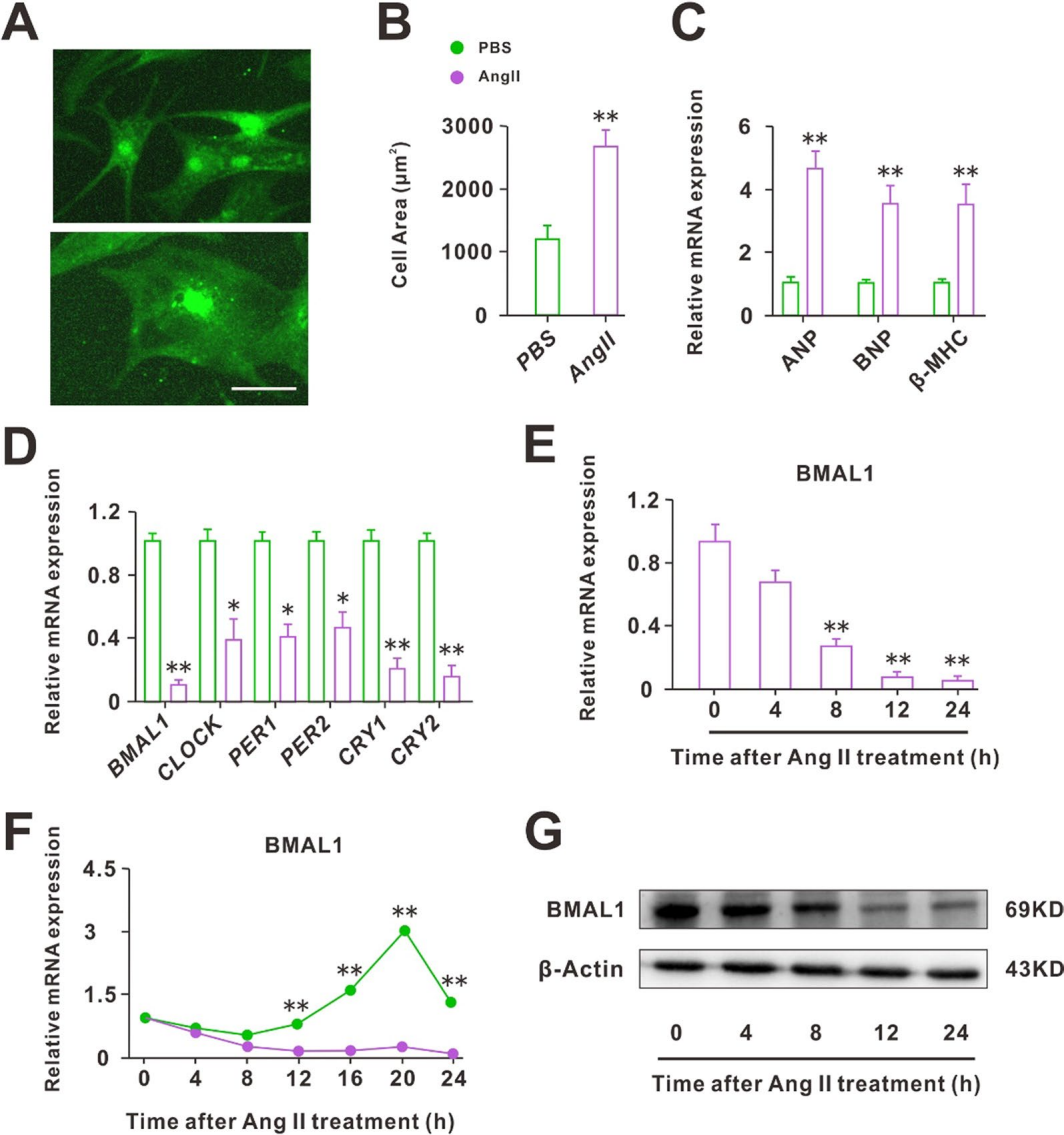
Cardiomyocyte hypertrophy led to the decrease of BMAL1 expression. **A** A-actinin staining of the cardiomyocytes before and after 24 h Ang II treatment (scale bar: 15 μm). **B** Statistical results of cell area by measuring random cells (n = 50 cells). **P < 0.01 versus PBS group. **C** Real-time PCR analyses of the hypertrophy markers atrial natriuretic peptide (ANP), brain natriuretic peptide (BNP), and β-myosin heavy chain (β-MHC) (n = 6). **P < 0.01 versus PBS group. **D** RT-PCR analyses of core clock genes, noting that the BMAL1 changes were most pronounced (n = 6). *P < 0.05 and **P < 0.01 versus PBS group. **E**, **G** RT-qPCR and WB analyses of BMAL1 expression treated with Ang II for 24 h (n = 6). **P < 0.01 versus Zero time. **F** RT-qPCR analysis of time-course mRNA expression of BMAL1 (n = 6). **P < 0.01 versus PBS group. All values are presented as the mean ± SEM

To investigate the role of BMAL1 in CH, we first induced NRCMs to produce CH using 24 h Ang II treatment. The volume and surface area of cells, as shown in Fig. 1A–C, increased significantly after 24 h Ang II treatment, as did the expression of CH markers (ANP, BNP, -MHC). Following that, the RT-PCR results suggested that CH may cause a decrease in the expression of core clock genes, with BMAL1 being the most affected (Fig. 1D). Furthermore, we identified the regulation of BMAL1 expression following a 24-h Ang II treatment. We found that Ang II treatment reduced the mRNA and protein expression levels of BMAL1 over time (Fig. 1E–G). More importantly, BMAL1 expression in normal cells followed a 24-h rhythm. The rhythm, however, disappeared when the cells became hypertrophic (Fig. 1F).

### BMAL1 restrained the Ang II-induced hypertrophy

**Fig. 2:**
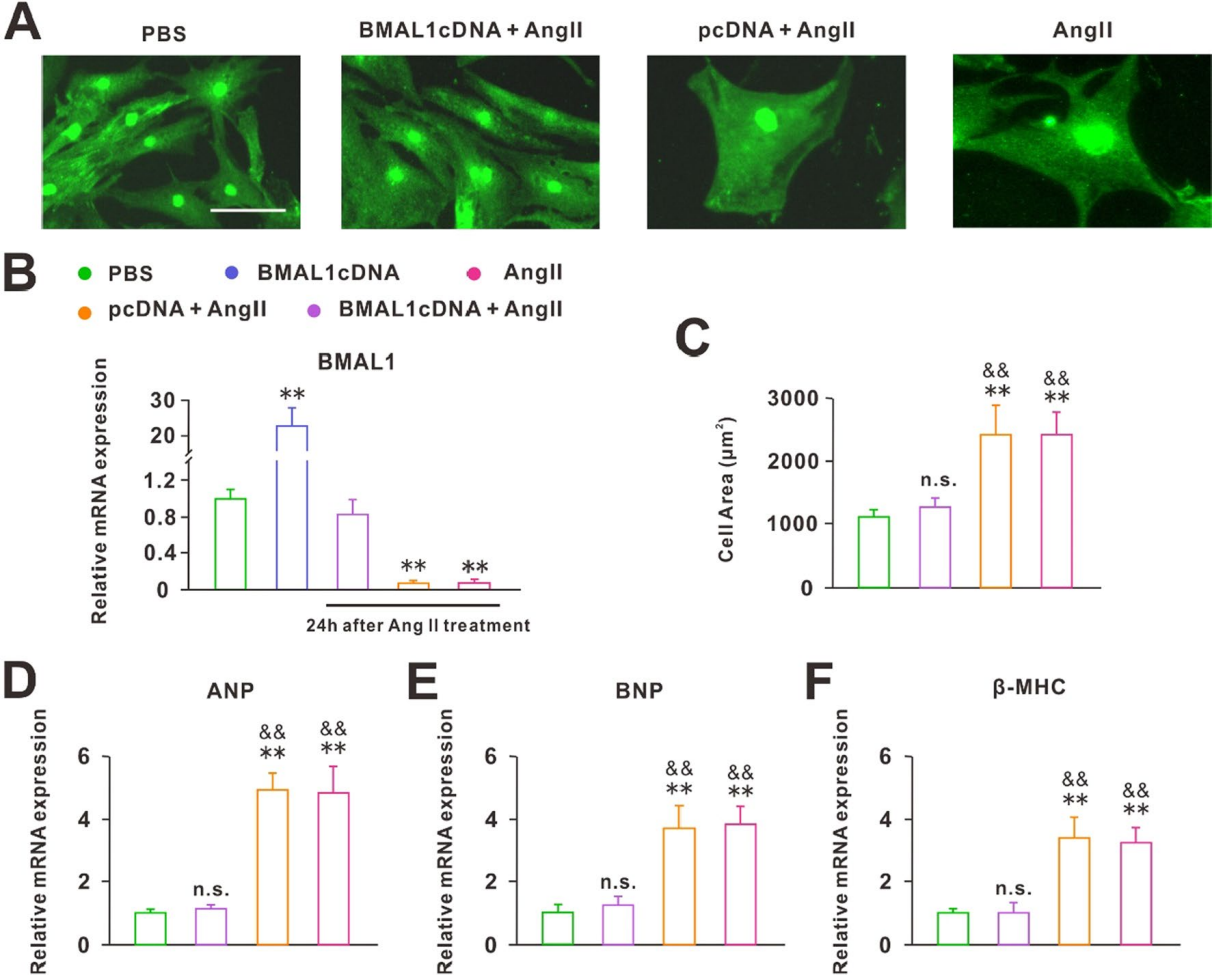
BMAL1 overexpression protected against Ang II-induced hypertrophy before Ang II treatment. **A** A-actinin staining of the normal cardiomyocytes or cardiomyocytes overexpressed BMAL1 (BMAL1cDNA) or negative controls (pcDNA) in response to Ang II for 24 h (scale bar: 15 μm). **B** RT-qPCR analyses of expression levels of BMAL1 in normal cardiomyocytes, BMAL1cDNA and BMAL1cDNA, pcDNA and normal cardiomyocytes teated with Ang II for 24 h (n = 6). **P < 0.01 versus PBS group. **C** The group data of cell area of different groups (n = 50). n.s., **P < 0.01 versus PBS group, ^&&^*P* < 0.01 versus BMAL1cDNA + Ang II group. **D**–**F** Real-time PCR analyses of the hypertrophy markers ANP, BNP and β-MHC in each group (n = 6). n.s. *P* > 0.1, **P < 0.01 versus PBS group, ^&&^*P* < 0.01 versus BMAL1cDNA + Ang II group. Group data presented by mean ± SEM

Because Ang II treatment reduced BMAL1 expression, we overexpressed BMAL1 (BMAL1cDNA) in the NRCMs prior to Ang II treatment to investigate its role in Ang II-induced hypertrophy. As shown in Fig. 2B, the expression of BMAL1 mRNA in BMAL1cDNA group was significantly higher than in normal cells. Although Ang II treatment also decreased the BMAL1 mRNA expression in the BMAL1cDNA cells, it was almost the same as that of normal cells. Corresponding to the BMAL1 mRNA expression, cardiomyocytes of the BMAL1cDNA group did not increase in size and cell surface area after Ang II treatment (Fig. 2A, C). In addition, the levels of ANP, BNP, and β-MHC did not change significantly in BMAL1cDNA cardiomyocytes treated with Ang II either (Fig. 2D–F). These results suggested that overexpression of BMAL1 effectively restrained the CH induced by Ang II.

**Fig. 3:**
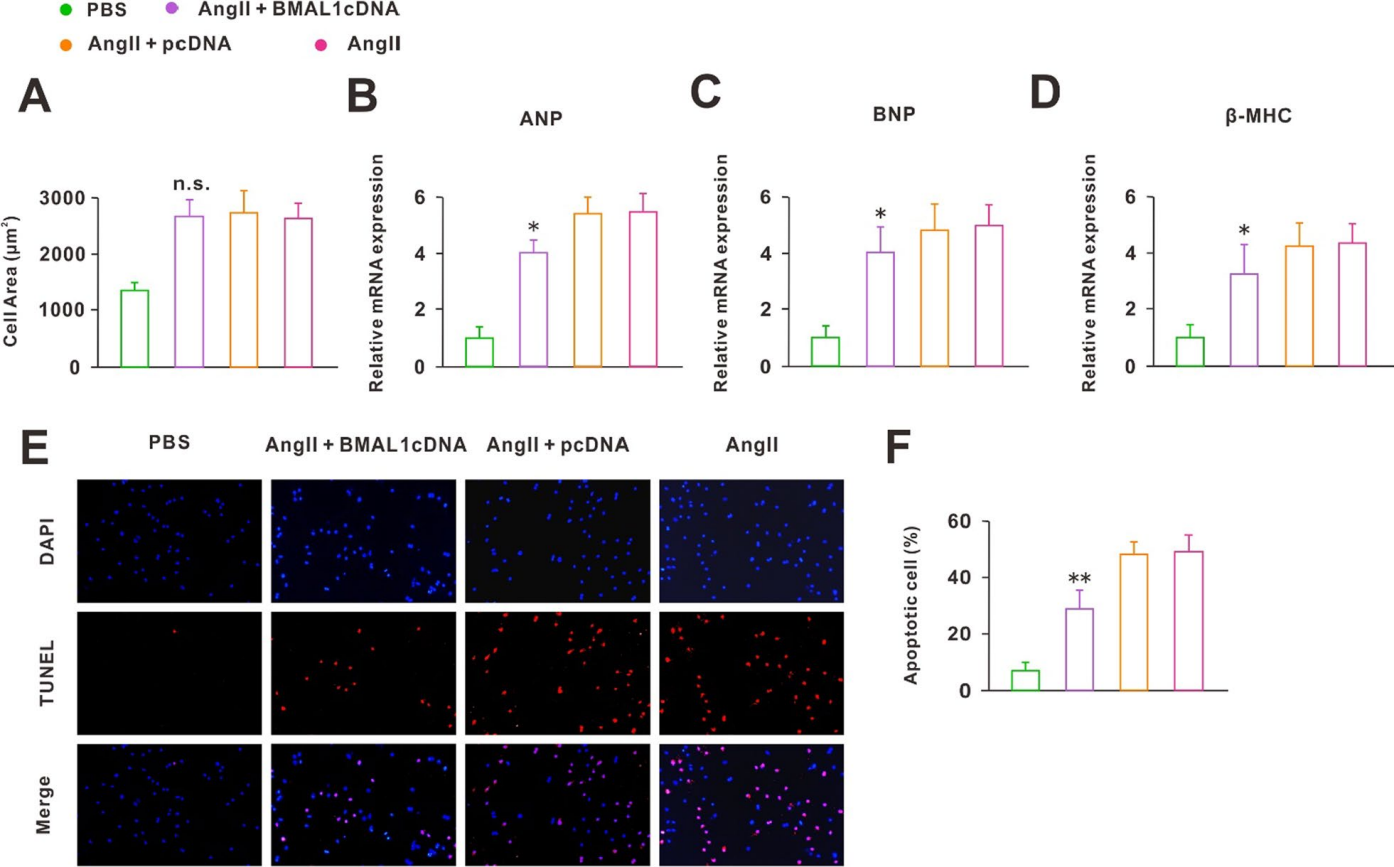
BMAL1 overexpression in hypertrophic cardiomyocytes did not rescue the hypertrophy symptom but decreased Ang II-induced cell apoptosis. **A** Statistical results of cell area by measuring random cells (n = 50 cells). n.s. *P* > 0.1 versus Ang II group. **B**–**D** Real-time PCR analyses of the hypertrophy markers ANP, BNP and β-MHC in each group (n = 6). *P < 0.05 versus Ang II group. **E** TUNEL assays. Pink: TUNEL-positive cells; Blue: DAPI. **F** Summarized data on the percentage of apoptotic cardiomyocytes in each group (n = 6). **P < 0.01 versus Ang II group. Group data presented by mean ± SEM

Overexpression of BMAL1 may protect against the hypertrophy before it occurs, but what about BMAL1 after the cardiomyocytes had already hypertrophy? Thus, NRCMs were treated with Ang II for 24 h before being infected with the adenoviruses encoding the BMAL1 CDS domain. We found that, when compared to the Ang II treatment group, overexpression of BMAL1 increased BMAL1 expression in hypertrophic cardiomyocytes (Additional file 1: Fig. S1), but overexpression of BMAL1 in hypertrophic cardiomyocytes did not rescue the hypertrophy symptoms (Fig. 3A). However, at the molecular level, the expression of three CH markers (ANP, BNP, and β-MHC) was reduced (Fig. 3B–D). Furthermore, we detected the apoptosis of cardiomyocytes by TUNEL assay. Interestingly, the results showed that overexpression of BMAL1 in hypertrophic cardiomyocytes significantly decreased Ang II-induced cell apoptosis (Fig. 3E, F). All the results above suggested that although BMAL1 did not alter the symptoms of CH once cardiomyocytes become hypertrophic, it was effective in reducing the apoptosis of hypertrophic cardiomyocytes.

**Fig. 4:**
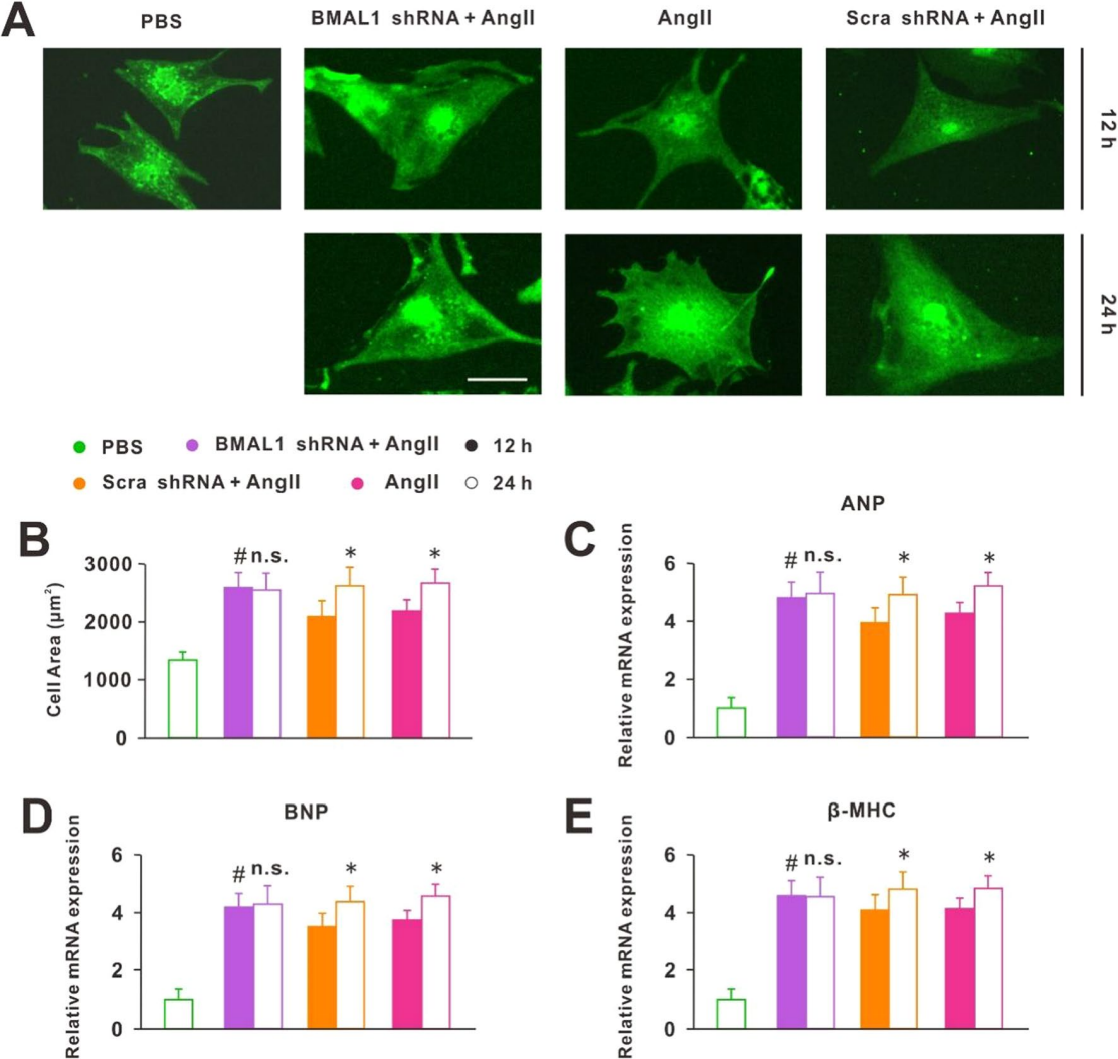
Knockdown of BMAL1 did not aggravate the symptom of CH but accelerated its development. **A** A-actinin staining of the cardiomyocytes or cardiomyocytes knocked down BMAL1 (BMAL1 shRNA + AngII) or negative controls (Scra shRNA + AngII) in response to Ang II for 12 h (top) and 24 h (bottom) (scale bar: 15 μm). **B** Statistical results of cell area by measuring random cells (n = 50 cells). n.s. *P* > 0.1, *P < 0.05 versus 12 h each group. ^#^*P* > 0.1 versus 24 h Ang II group. **C**–**E** Real-time PCR analyses of the hypertrophy markers ANP, BNP and β-MHC in each group (n = 6). n.s. *P* > 0.1, *P < 0.05 versus 12 h each group. ^#^*P* > 0.1 versus 24 h Ang II group. Group data presented by mean ± SEM

In addition, we knocked down BMAL1 through transfection with BMAL1 shRNA, the BMAL1 expression in each group was shown in Additional file 1: Fig. S2. After 24 h Ang II treatment, there was no significant difference in cell size (Fig. 4A), surface area (Fig. 4B) and levels of ANP, BNP, and β-MHC (Fig. 4C–E) between BMAL1 knocked down and normal cells. It suggested that knockdown of BMAL1 could not aggravate hypertrophy of cardiomyocytes. However, we found that the normal cells were less hypertrophic after 12 h Ang II treatment than 24 h (Fig. 4A, B), while BMAL1 knocked down cells showed the same cell size between Ang II treatment for 12 h and 24 h (Fig. 4A, B). Moreover, the PCR results of three CH markers (ANP, BNP and, β-MHC) were consistent with the immunofluorescence analysis (Fig. 4C–E). These results suggested that knockdown of BMAL1 did not aggravate the symptom of CH, but would accelerate its development.

### BMAL1 restrained cardiac oxidative stress and boosted myocardial autophagy

**Fig. 5:**
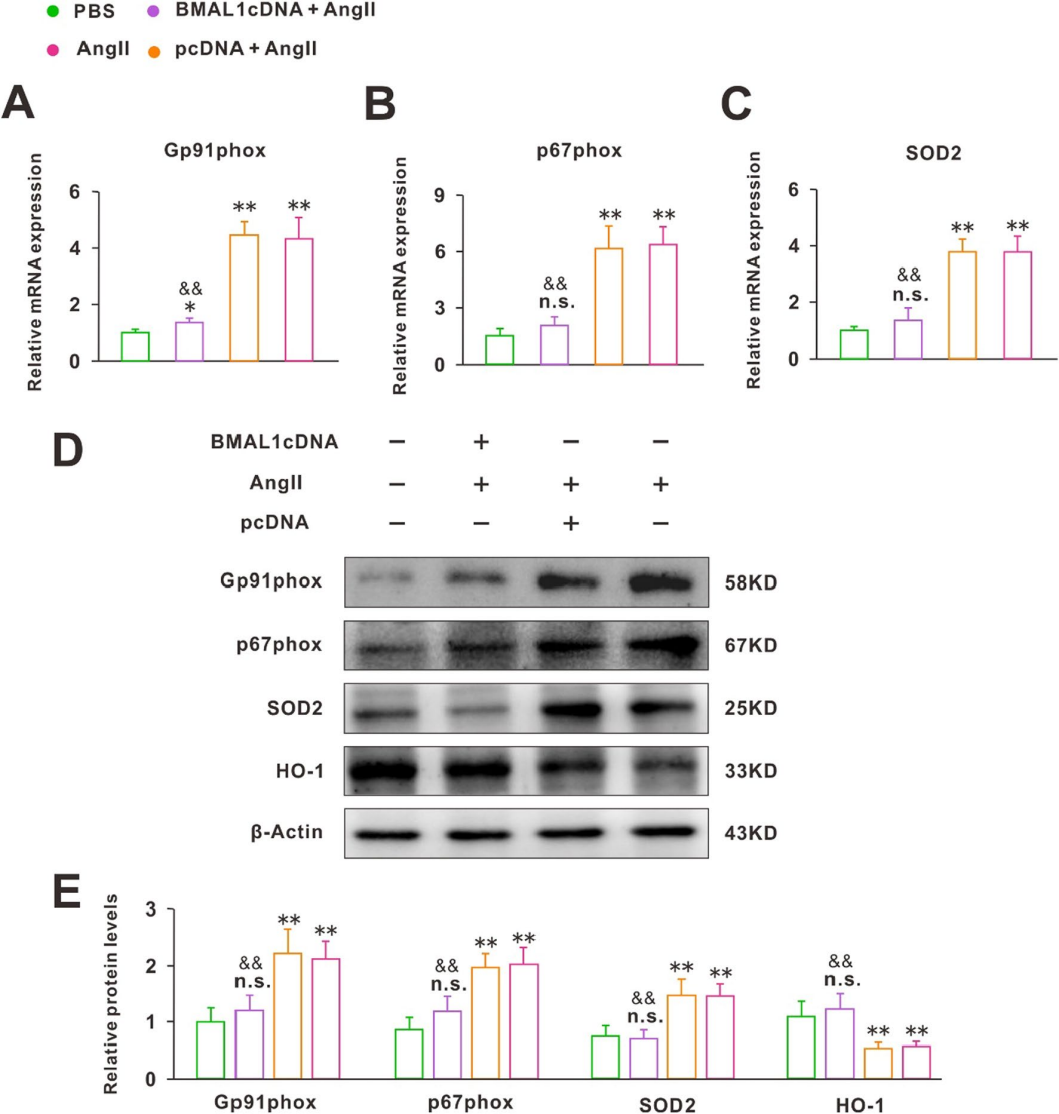
BMAL1 restrained cardiac oxidative stress in hypertrophic cardiomyocytes induced by Ang II. **A**–**C** Real-time PCR analyses of the NADPH oxidase subunits gp91phox, p67phox and SOD2 (n = 6). n.s. *P* > 0.1, *P < 0.05, **P < 0.01 versus PBS group, ^&&^*P* < 0.01 versus Ang II group. **D**, **E** Immunoblotting and quantification analyses of the protein levels of gp91phox, p67phox, SOD2 and HO-1 in each group (n = 6). The red arrow identified the target protein bands. n.s. *P* > 0.1, **P < 0.01 versus PBS group, ^&&^*P* < 0.01 versus Ang II group. Group data presented by mean ± SEM

Studies had shown that oxidative stress and inflammation were demonstrated to contribute to Ang II-induced CH [19][19]. Thus, we evaluated the antioxidant effects of BMAL1 by assessing the transcription levels of the NADPH oxidase subunits gp91phox, p67phox, SOD2, and the haem oxygenase 1 (HO-1). The results showed that compared with the normal cells, the cardiac mRNA levels of gp91phox, p67phox and SOD2 were all significantly lower in the hypertrophic cardiomyocytes that overexpressed BMAL1 after 24 h Ang II treatment (Fig. 5A–C). Protein expression was detected by WB showed similar alterations as mRNA levels (Fig. 5D, E). In addition, overexpression of BMAL1 restored HO1 (Fig. 5D, E).

**Fig. 6:**
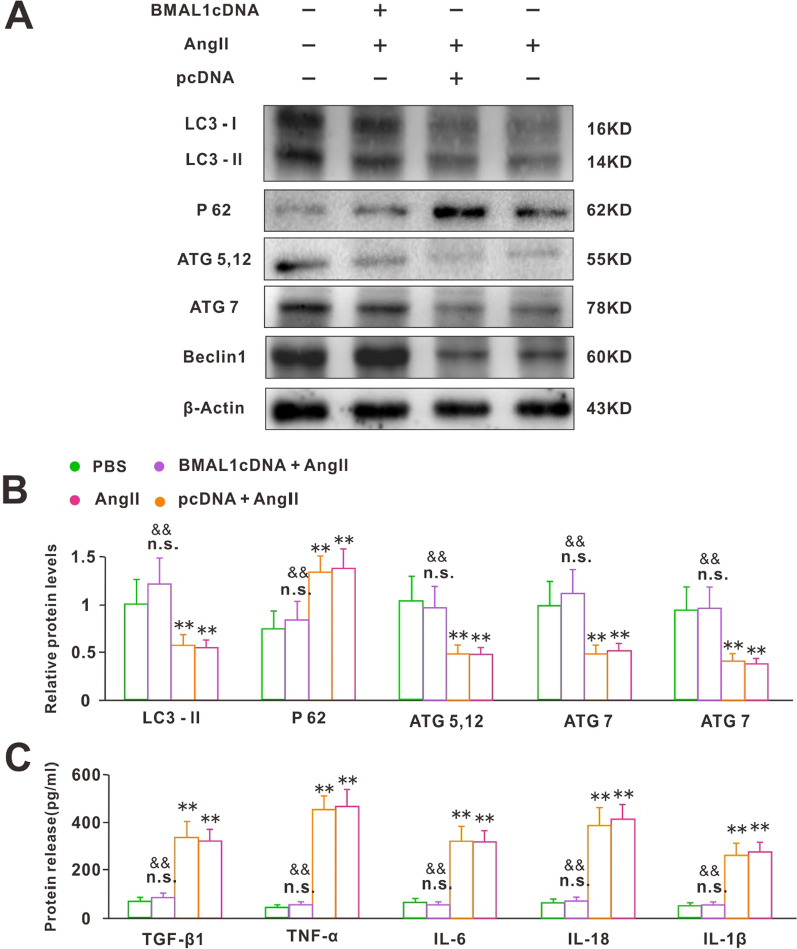
BMAL1 boosted myocardial autophagy in hypertrophic cardiomyocytes induced by Ang II. **A** Raw data of proteins expression of a panel of autophagy-related genes detected by WB. **B** Quantification analyses of the protein levels of LC3I/LC3II, P62, ATG5-12, ATG7 and Beclin1 in each group (n = 6). n.s. *P* > 0.1, **P < 0.01 versus PBS group, ^&^*P* < 0.05, ^&&^*P* < 0.01 versus Ang II group. Group data presented by mean ± SEM. **C** The release of cytokines in cell-free supernatants, including TGF-β1, TNF-α, IL-6, IL-18 and IL-1β in each group (n = 6). n.s. *P* > 0.1, **P < 0.01 versus PBS group, ^&^*P* < 0.05, ^&&^*P* < 0.01 versus Ang II group. Group data presented by mean ± SEM

Impaired autophagy could lead to exacerbated cardiac oxidative stress [17]. Autophagic activities were analyzed by immunoblotting of a panel of autophagy-related genes in normal and BMAL1 over-expressed cells after 24 h Ang II treatment. In BMAL1 over-expressed cells, LC3II expression was significantly increased, with increased ATG5-12, ATG7, and Beclin1, but decreased accumulation of P62 (Fig. 6A, B). These results suggested that overexpression of BMAL1 would enhance the autophagic function of cardiomyocytes. Moreover, Ang II was well known to be a trigger of cardiac fibrosis mediated by cardiac fibroblasts [21]. We further tested the release of cytokines in cell-free supernatants, which led to the activation of cardiac fibroblasts, including TGF-β1, TNF-α, IL-6, IL-18, and IL-1β. As shown in Fig. 6C, BMAL1 overexpression significantly decreased the concentrations of these cytokines in cell-free supernatants after 24 h Ang II treatment. These findings suggested that Bmal1 overexpression may protect against Ang II-induced cardiac fibrosis.

## Discussion

Pathologic myocardial hypertrophy was a major risk factor for heart failure and was closely related to heart progressive dysfunction. At present, many mechanisms had been proved to be related to the occurrence and development of CH. However, people living irregular life had a higher risk of high blood pressure, inflammation, and heart damage, all of which were becoming more common in modern society [22]. Moreover, studies have shown that Circadian Clock Regulation is associated with a variety of pathologies, including insomnia, senility, cancer, metabolic syndrome, immune system disorders, and cardiovascular disease [16]. Especially, mutations in BMAL1 or its partner CLOCK could lead to cardiovascular disease. The CLOCK Δ19 mice showed hypertrophy, decreased contractility, and reduced myogenic reactivity [23]. Specific mutations in the CLOCK gene resulted in systolic dysfunction [24]. Similarly, the absence of BMAL1 led to age-related dilated cardiomyopathy [15]. In addition, mice with cardiomyocyte-specific BMAL1 knock-out developed dilated heart disease as they grew older [25]. In the BMAL1 knockout human embryonic stem cell (hESC) model, BMAL1 deficient hESC-derived cardiomyocytes exhibited typical phenotypes of dilated cardiomyopathy including attenuated contractility, calcium dysregulation, and disorganized myofilaments [16].

Notably, all the study above focused on the consequences of circadian clock gene deletions on normal animals or cells. However, in pathological conditions, the role of circadian clock genes was not clear. Our work suggested that the core clock gene BMAL1 was closely associated with Ang II-induced CH. First, we examined the expression of circadian clock genes in hypertrophic cardiomyocytes induced by Ang II and found that the expression of all genes decreased, with the greatest change in the expression of BMAL1 (Fig. 1D–G). In addition, after Ang II treatment, the 24-h rhythm of BMAL1 was lost (Fig. 1F). These results suggested that CH disrupted the physiological state of clock genes, especially BMAL1. So whether the recovery of BMAL1 expression would improve the symptoms of CH? Next, we overexpressed BMAL1 before and after Ang II treatment, separately. The results showed that before Ang II treatment, BMAL1 overexpression protected against Ang II-induced CH. On the contrary, when the cardiomyocytes became hypertrophic, the increased expression of BMAL1 did not save the cells from hypertrophy. However, under this situation, overexpression of BMAL1 significantly reduced the apoptosis of hypertrophic cardiomyocytes (Fig. 3). In addition, although knockdown of BMAL1 did not influence the degree of CH, it accelerated the development of CH (Fig. 4). This effects of BMAL1 on Ang II-induced CH, on the one hand, suggested that people or animals with dysrhythmia are more likely to suffer from CH, on the other hand, BMAL1 might be used as an ideal target of new drugs for the prevention and treatment of patients with CH.

In the process of Ang II induced CH, Ang II activated NADPH to produce reactive oxygen species (Ros). Ros finally regulated genes related to hypertrophy such as ANF, BNP, and β-MHC, which led to the increase in protein production in cardiomyocytes [17]. Thus, we assessed the transcription levels of the NADPH oxidase subunits gp91phox, p67phox, and SOD2. BMAL1 overexpression significantly inhibited the expression of these components. Correspondingly, the expression of HO1, which had an antioxidation effect, was increased (Fig. 5). These results suggested that BMAL1 protected against CH induced by Ang II through resisting oxidative damage. Autophagy is one of the important metabolic pathways of eukaryotic cells. Numerous studies had shown that Ros could activate autophagy [26–28]. Disruption of autophagy has proved to be related to several cardiovascular disorders, such as myocardial infarction, cardiomyopathies, atherosclerosis, and cardiotoxicity [29]. Similarly, our WB and PCR results showed that the transcription levels of genes associated with autophagy significantly changed in hypertrophic cardiomyocytes. However, it was worth noting that BMAL1 overexpression significantly resisted the inhibition of Ang II on autophagy (Fig. 6). Previous studies have found that autophagy cleared mitochondria, endoplasmic reticulum, peroxisome, and proteins damaged by oxidative stress, and prolonged cardiomyocytes’ lives [30]. It meant the effect of BMAL1 on autophagy might be responsible for its antioxidant properties.

Ang II was well known to be a trigger of cardiac fibrosis mediated by cardiac fibroblasts [21]. And Ang II-induced CH and fibrosis were blocked in TGF-β1 knockout mice. TGF-β is a strong inducer of the differentiation of fibroblasts to myofibroblasts [31]. We first tested the concentration of TGF-β1 in the cell-free supernatants after 24 h Ang II treatment. The results showed that BMAL1 overexpression effectively resisted the increase of TGF-β1 induced by Ang II (Fig. 6C). After injury or during aging, fibroblasts are activated by the stimulation of inflammatory cytokines [21]. Similarly, BMAL1 overexpression had the same effects on the inflammatory cytokines TGF-β1, TNF-α, IL-6, IL-18, and IL-1β, which would lead to the activation of fibroblasts (Fig. 6C). Altogether, these results suggested that BMAL1 overexpression inhibited the inflammatory reaction and prevented myocardial fibrosis induced by Ang II. Thus, in the heart, BMAL1 overexpression might protect against cardiac fibrosis. Interestingly, BMAL1 was required for the TGF-β1-induced signaling transduction and pro-fibrotic activities in the lung [32]. It indicated that the mechanisms of BMAL1 effects on fibrosis in different tissues were different and worthy of further study.

## Conclusions

Our results showed that overexpression of BMAL1 effectively resisted CH induced by Ang II. This protective effect was achieved by enhancing autophagy to restrain oxidative stress. Our findings provided a novel potential target for the treatment of CH.

## Supplementary Information

**Additional file 1**.** Figure S1**. RT-qPCR analyses of BMAL1 mRNA expression in each group. PBS, control group. Ang II + BMAL1cDNA, BMAL1 overexpression was performed after cardiomyocytes having become hypertrophic induced by Ang II. Ang II + pcDNA, negative controls group. Ang II, normal cardiomyocytes treated with Ang II. **P < 0.01 vs. Ang II. Data are represented as mean ± SEM. **Figure S2**. RT-qPCR analyses of BMAL1 mRNA expression in each group. PBS, control group. BMAL1 shRNA, BMAL1 knockdown group. BMAL1 shRNA + AngII, BMAL1 knockdown and treated with AngII for 24h, Scra shRNA + AngII, negative controls group. Ang II, normal cardiomyocytes treated with Ang II. **P < 0.01 vs. PBS group, n.s. P > 0.1 vs. BMAL1 shRNA + Ang II group, # P > 0.1 vs. Ang II group. Data are represented as mean ± SEM.** Supplementary Table 1**. Lists of primer sequences for qPCR analysis, and shRNA oligonucleotides for gene knockdown.

## Data Availability

The data set used and/ or analyzed during the current study is available from the corresponding author upon reasonable request.
