# Supplementary material for: BMAL1 plays a critical role in the protection against cardiac hypertrophy through autophagy in vitro
Source: BMC Cardiovasc Disord. 2022 Aug 22;22:381. doi: 10.1186/s12872-022-02822-3 (PMC9396899; doi:10.1186/s12872-022-02822-3)
Supplement: Supplementary file 1 — Additional file 1. Figure S1. RT-qPCR analyses of BMAL1 mRNA expression in each group. PBS, control group. Ang II + BMAL1cDNA, BMAL1 overexpression was performed after cardiomyocytes having become hypertrophic induced by Ang II. Ang II + pcDNA, negative controls group. Ang II, normal cardiomyocytes treated with Ang II. **P < 0.01 vs. Ang II. Data are represented as mean ± SEM. Figure S2. RT-qPCR analyses of BMAL1 mRNA expression in each group. PBS, control group. BMAL1 shRNA, BMAL1 knockdown group. BMAL1 shRNA + AngII, BMAL1 knockdown and treated with AngII for 24h, Scra shRNA + AngII, negative controls group. Ang II, normal cardiomyocytes treated with Ang II. **P < 0.01 vs. PBS group, n.s. P > 0.1 vs. BMAL1 shRNA + Ang II group, # P > 0.1 vs. Ang II group. Data are represented as mean ± SEM. Supplementary Table 1. Lists of primer sequences for qPCR analysis, and shRNA oligonucleotides for gene knockdown. [file 12872_2022_2822_MOESM1_ESM.docx]

**Fig. S1**

**
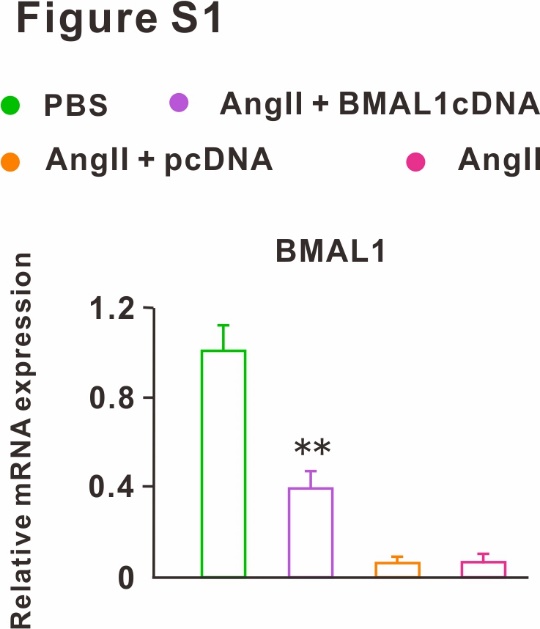
**

**Figure S1.** RT-qPCR analyses of BMAL1 mRNA expression in each group. PBS, control group. Ang Ⅱ + BMAL1cDNA, BMAL1 overexpression was performed after cardiomyocytes having become hypertrophic induced by Ang Ⅱ. Ang Ⅱ + pcDNA, negative controls group. Ang Ⅱ, normal cardiomyocytes treated with Ang Ⅱ. ***P* < 0.01 *vs.* Ang Ⅱ. Data are represented as mean ± SEM.

**Fig. S2**

**
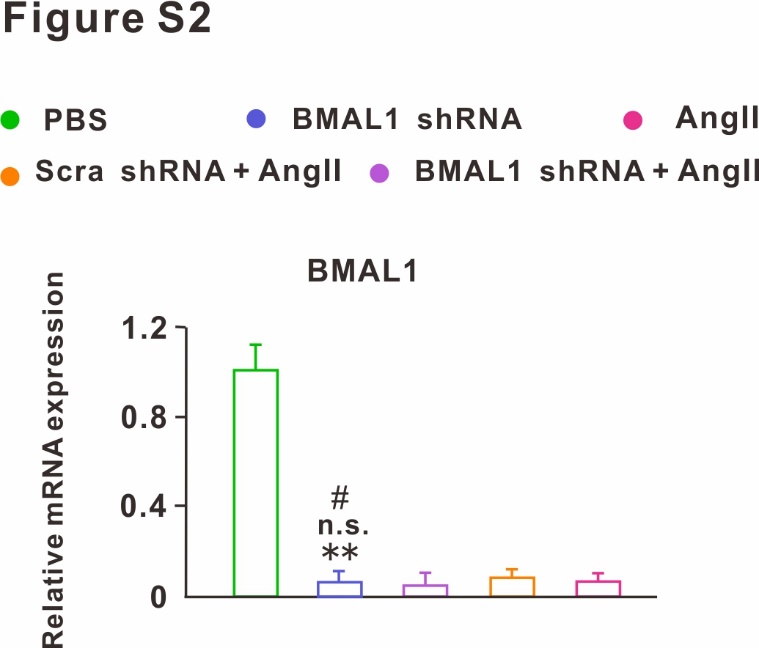
**

**Figure S2.** RT-qPCR analyses of BMAL1 mRNA expression in each group. PBS, control group. BMAL1 shRNA, BMAL1 knockdown group. BMAL1 shRNA + AngII, BMAL1 knockdown and treated with AngII for 24h, Scra shRNA + AngII, negative controls group. Ang Ⅱ, normal cardiomyocytes treated with Ang Ⅱ. ***P* < 0.01 *vs.* PBS group, n.s. *P* > 0.1 *vs.* BMAL1 shRNA + AngII group, # *P* > 0.1 *vs.* Ang II group. Data are represented as mean ± SEM.

**Supplementary Table 1** Lists of primer sequences for qPCR analysis, and shRNA oligonucleotides for gene knockdown.

| **List of Primers used for qPCR analysis.** | | | |
| --- | --- | --- | --- |
| **Genes** | **Forward Primer Sequences (5’-3’)** | | **Reverse Primer Sequences (5’-3’)** |
| *β-Actin* | AGCCATGTACGTAGCCATCC | | CTCTCAGCTGTGGTGGTGAA |
| *Bmal1* | TGGAGGGACTCCAGACATTC | | TGGGACTACTTGATCCTTGG |
| *clock* | CACTCTCACAGCCCCACTGTAC | | CCCCACAAGCTACAGGAGCAGT |
| *Per1* | AACGGGATGTGTTTCGGGGTGC | | AGGACCTCCTCTGATTCGGCAG |
| *Per2* | TGATCGAGACGCCTGTGCTCGT | | CTCCACGGGTTGATGAAGCTGG |
| *Cry1* | AGCGCAGGTGTCGGTTATGAGC | | ATAGACGCAGCGGATGGTGTCG |
| *Cry2* | TGGGCATCAACCGATGGAG | | CCCATTCCTTGAACAGCCTTG |
| *β-MHC* | TTTGATGTGCTGGGCTTCAC | | TGACATACTCGTTGCCCACT |
| *ANP* | GACGCCCTCCGATGTGAAAG | | GGCTCTGTTACTGCTTAGTTCAA |
| *BNP* | CAGAAGGTGCTGCCCCAGATG | | GACTGCGCCGATCCGGTC |
| *gp91phox* | GAATCTCAGGCCAATCACTT | | TGGTCTTGAACTCGTTATCCC |
| *p67phox* | AGCAGAAGAGCAGTTAGCATTGG | | TGCTTTCCATGGCCTTGTC |
| *SOD2* | CTGAGGAGAGCAGCGGTCGT | | CTTGGCCAGCGCCTCGTGGT |
| **The sequences of shRNA oligonucleotides for gene knockdown** | | | |
|  | **Sequences (5’-3’)** | | |
| *scramble shRNA* | Top strand | TGCTGAAATGTACTGCGCGTGGAGACGTTTTGGCCACTGACTGACGTCTCCACGCAGTACATTT | |
|  | Bottom strand | CCTGAAATGTACTGCGTGGAGACGTCAGTCAGTGGCCAAAACGTCTCCACGCGCAGTACATTTC | |
| *Bmal1 shRNA* | Top strand | TGCTGAATGTTGGCTTGTAGTTTGCTGTTTTGGCCACTGACTGACAGCAAACTAAGCCAACATT | |
|  | Bottom strand | CCTGAATGTTGGCTTAGTTTGCTGTCAGTCAGTGGCCAAAACAGCAAACTACAAGCCAACATTC | |

**Raw Data of WB**

**
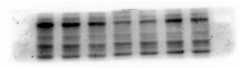
**

**
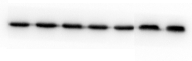
 Fig. 1G BMAL1**

**Fig. 1G β-Actin**

**
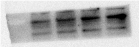
**

**Fig. 5D p67phox**

**
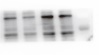
**

**Fig. 5D SOD2**

**
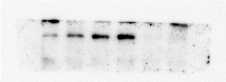
**

**Fig. 5D Gp91phox**

**
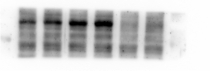
**

**Fig. 5D HO-1**

**
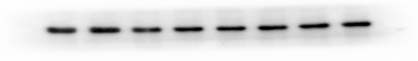
**

**Fig. 5D β-Actin**

**
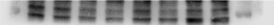
**

**Fig. 6A** **LC3 – I and LC3-Ⅱ**

**
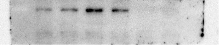
**

**Fig. 6A p
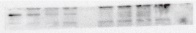
62**

**Fig. 6A ATG 5, 12**

**
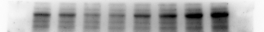
**

**Fig. 6A ATG 7**

**
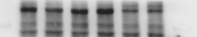
**

**Fig. 6A beclin1**

**
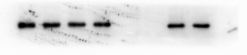
**

**Fig. 6A G β-Actin**
